# Supplementary material for: Infodemiologists Beware: Recent Changes to the Google Health Trends API Result in Incomparable Data as of 1 January 2022
Source: Int J Environ Res Public Health. 2022 Nov 21;19(22):15396. doi: 10.3390/ijerph192215396 (PMC9692853; doi:10.3390/ijerph192215396)
Supplement: Supplementary file 1 [file ijerph-19-15396-s001.zip › ijerph-1960959-supplementary.pdf]

Table S1. List of searched terms included in this investigation. It should be noted that the presented supplementary figures' numbers do not correspond with the order that the keywords are presented here.

Freebase ID / Google Knowledge Graph Identifier	English Term
/m/0pzy	Acacia
/m/02015n	Acetazolamide
/m/0bsk8	Acetic acid
/m/0dv67	Acetylcholine
/m/01_ds3	Acetylcysteine
/m/0t2t	Acetylene
/m/02xbjr	Aciclovir
/m/02378v	Activated carbon
/g/11ll0qhr92	Ad26.COV2.S
/m/03qkby	Adapalene
/g/12384rxk	Adrenaline
/m/0fx47	Adrenocorticotropic hormone
/m/01sbwk	Agaricus bisporus
/m/02kbv4h	Aldosterone
/m/03n1kq	Alkaline phosphatase
/m/01ls3h	Allopurinol
/m/01k9cw	Alprazolam
/m/027vj2v	Aluminium
/m/04wrbh	Aluminium chloride
/m/011w2f	Aluminium oxide
/m/0pbc	Amber
/m/0m_j	Amino acid
/m/02sff3	Amitriptyline
/m/02_0mv	Amlodipine
/m/0p6_	Ammonia
/m/01qwgy	Ammonium chloride
/m/011z0	Amoxicillin
/m/01rr9y	Aniline
/m/027s2zq	Antimony
/m/0k8z	Apple
/m/05253_m	Apple cider vinegar
/m/0fnfy	Apricot
/m/0b1p9p	Argan oil
/m/0y44	Ascorbic acid
/m/0cjs7	Asparagus
/m/0qkc	Aspirin
/m/034yzx	Atenolol
/m/02y_rs	Atorvastatin
/m/012fbv	Atropine
/m/01645p	Avocado
/m/032n3z	Azithromycin
/m/09qck	Banana
/m/09gtx	Barley
/m/01yt8c	Basil
/m/01bzn	BCG vaccine
/m/01fp9	Bean

/m/0971v	Beef
/m/0bxq8	Beeswax
/m/0jg57	Bell pepper
/m/01fr51	Bentonite
/m/01bbg	Benzoic acid
/m/03h2f_	Benzoyl peroxide
/m/057xmt0	Benzylpenicillin
/m/057fff	Betamethasone
/m/0f4ml	Biotin
/m/0994n3	Bisoprolol
/m/0139zp	Black pepper
/m/0ftbs	Blueberry
/m/0hdcp	Borax
/m/0hd8m	Boric acid
/m/025svlc	Boron
/g/120zt_4k	Botulinum toxin
/m/09twn9b	Broccoli
/m/025tksd	Cadmium
/m/01_7l	Caffeine
/m/025tkqy	Calcium
/m/0c5pb	Calcium carbonate
/m/012w7x	Calcium oxide
/m/04klcx	Cannabidiol
/m/01_y8	Carbamazepine
/m/01s7k	Carbon dioxide
/m/01v1v	Carbon monoxide
/g/11bc5yhfnk	Carrot
/m/0m7j1	Casein
/m/0h259	Cashew
/m/0174xf	Castor oil
/m/0cqrj	Catfish
/m/03x2w7	Ceftriaxone
/m/01xj_	Celery
/m/03t7zn	Cetirizine
/m/011bc8hg	Chamomile
/m/0f8sw	Cherry
/m/09b5t	Chicken
/m/0fqtq	Chickpea
/m/01whr	Chloramphenicol
/m/03n352	Chlorhexidine
/m/0hjfy	Chloride
/m/025tkqp	Chlorine
/m/0l80n	Chloroform
/m/05c7gk	Chloroquine
/m/0133fz	Chlorpromazine
/m/01w_3	Cholesterol
/m/025tkr6	Chromium
/m/0f0ch	Cinnamon
/m/01zfw	Ciprofloxacin
/m/0f7gf	Citric acid
/m/02z4qr	Clindamycin

/m/044r6z	Clomifene
/m/035wxl	Clonazepam
/m/02svfj	Clopidogrel
/m/05l1rk	Clotrimazole
/g/121hrbw_	Clove
/m/0f4k5	Cobalamin
/m/025tkrf	Cobalt
/m/0256b	Cocaine
/m/03mjm_	Cocoa butter
/m/0djtd	Coconut
/m/01jszz	Coconut oil
/m/01f4g6	Cod liver oil
/m/0256v	Codeine
/m/0p3rn	Coenzyme Q10
/m/05bzrs	Coffee bean
/m/01_yv2	Colchicine
/m/025rsfk	Copper
/g/120vb81q	Cotton
/m/020dp	Cranberry
/m/01wyhy	Creatine
/m/0hdqh	Creatinine
/m/015x4r	Cucumber
/m/01qs2	Cumin
/m/02x9wcz	Dapagliflozin
/m/01x1z5	Dexamethasone
/m/01j3v_	Diazepam
/m/01_94h	Diclofenac
/m/01xc5b	Digoxin
/m/0256qn	Diphenhydramine
/m/0c_3s	Dopamine
/m/030md9	Doxycycline
/m/033cnk	Egg
/m/01zvtl	Egg white
/m/0cdk2	Eggplant
/m/018_cj	Ephedrine
/m/02r51	Erythromycin
/m/01fwr3	Erythropoietin
/m/039ztp	Esomeprazole
/m/01h2ty	Estradiol
/m/02qvz	Ethanol
/m/04khtxp	Ethylenediaminetetraacetic acid
/m/017dqv	Fennel
/m/011w3r	Fentanyl
/m/043nyj	Fig
/m/04lr99	Finasteride
/m/03y0gb	Fish oil
/m/03dwr5	Fluconazole
/m/025rqc3	Fluorine
/m/0bqx0	Fluoxetine
/m/02kb_jm	Folate
/m/0h3r1	Formaldehyde

/m/0d9t0	Fructose
/m/02fb0h	Furosemide
/m/02fvfb	Gabapentin
/m/0dbrl	Garlic
/m/03fz3	Gelatin
/m/01lrtb	Gentamicin
/g/121d6gs3	Ginger
/m/0htd3	Ginkgo
/m/029x39	Ginseng
/m/079695	Glibenclamide
/m/01l2q7	Glucagon
/m/02kb_m_	Glucose
/m/0k_xf	Glutathione
/m/01781c	Glycerol
/m/02kc008	Glycine
/m/075w2f	Glycolic acid
/m/01q_vm	Glyphosate
/g/122hcm0p	Goat milk
/m/025rs2z	Gold
/m/0dbvp	Goose
/m/0388q	Grape
/m/0hqkz	Grapefruit
/m/0hqvklw	Growth hormone
/m/0199v_	Haloperidol
/m/06wcblr	Hazelnut
/m/025rt79	Helium
/m/01jjpv	Heparin
/m/02z0_x8	Hepatitis B vaccine
/m/03n5b	Heroin
/m/0d7j9	Herring
/m/0238z7	Histamine
/g/11fj7lyvsb	Honey
/m/03qjz	Hops
/m/0cdkl	Horseradish
/m/01rtx2	Human chorionic gonadotropin
/m/04ldlf	Hyaluronic acid
/m/0dc0x	Hydrochloric acid
/m/02nsfz	Hydrochlorothiazide
/g/11c53146q3	Hydrocortisone
/m/02h9byz	Hydrogen
/m/03q_p	Hydrogen peroxide
/m/05pqnv	Hydroquinone
/m/03km2	Hydroxide
/m/071ws0	Hydroxychloroquine
/m/03jdr1	Hypochlorite
/m/014d3g	Ibuprofen
/m/03ztdf	Imipramine
/m/02dpgh	Indole
/m/04h8mb	Indomethacin
/m/02kyfb	Iodide
/m/025rw19	Iron

/m/01fmj4	Iron(III) oxide
/m/03zd_	Isopropyl alcohol
/m/01hgfy	Isotretinoin
/m/047j8m	Ivermectin
/m/081mv8	Jojoba oil
/m/04b5r	Ketamine
/m/03bxlz	Ketoconazole
/m/04f8p	Kiwifruit
/m/0176jz	Lactic acid
/m/04qlf	Lactose
/m/03b15z	Lamivudine
/m/01zww4	Lamotrigine
/m/02lj4d	Lecithin
/m/09k_b	Lemon
/m/017kh0	Lentil
/m/08924w	Letrozole
/m/0fqlj	Lettuce
/m/02mx2z	Levofloxacin
/m/031zs3	Levonorgestrel
/m/05h3j6	Levothyroxine
/m/01rml9	Lidocaine
/m/01k_hc	Liquorice
/m/0cjq5	Lobsters
/m/01qydv	Loperamide
/m/0dgq98	Lopinavir
/m/025s3l	Loratadine
/m/072klj	Losartan
/m/01kysw	Luteinizing hormone
/m/04g9r	Lysergic acid diethylamide
/m/0h1vz	Lysine
/m/025s0s0	Magnesium
/m/02q788	Magnesium chloride
/m/01bfyf	Magnesium hydroxide
/m/015jzg	Magnesium oxide
/m/01kmqg	Magnesium sulfate
/m/01f5cb	Maltose
/m/025s0zp	Manganese
/m/03zf_l	Mannitol
/m/01pxjm	Melatonin
/m/01v03x	Menthol
/m/01lmkt	Metformin
/m/0gt5b	Methamphetamine
/m/04ydh	Methane
/m/01q2jc	Methotrexate
/m/01jmw4	Methylene blue
/m/01d65r	Methylphenidate
/m/05zwj8	Methylprednisolone
/m/021xx2	Metoclopramide
/m/01trbt	Metronidazole
/m/03npzj	Miconazole
/m/02_7lx	Midazolam

/m/018svj	Mifepristone
/m/04zpv	Milk
/m/073ynx	Milk thistle
/m/01f3_7	Mineral oil
/m/027hm4	Minoxidil
/m/055pr	Morphine
/m/016gq4	Morus alba
/m/019pht	Mustard seed
/m/0n_fl	Myrrh
/m/09gvd	Niacin
/m/025s5r7	Nicotinamide
/m/09jrx	Nicotine
/m/035bjg	Nifedipine
/m/05d92	Nitrate
/m/05fmf	Nitric acid
/m/01j5q2	Nitric oxide
/m/025s4ld	Nitrogen
/m/05dkv	Nitroglycerin
/m/09bml	Nitrous oxide
/m/09lnn	Nutmeg
/m/0cl2v	Oat
/m/025s18p	Okra
/m/05ml4	Olive oil
/m/05nkr	Omega 3
/m/02gbr5	Omeprazole
/m/04b72x	Ondansetron
/m/0dj75	Onion
/m/05pg4	Opium
/m/05kw_	Oregano
/m/025s6bf	Oxygen
/m/02n6zm	Oxytetracycline
/m/01gfsk	Oxytocin
/m/0_cp5	Oyster
/m/025s7xf	Palladium
/m/0fsx5	Palm oil
/m/0g4fr	Papaya
/m/0svg9	Paprika
/m/0lbt3	Paracetamol
/m/0264dt	Parathyroid hormone
/m/05s28	Parsley
/m/0dj6p	Peach
/m/0fg0m	Peanut
/m/061_f	Pear
/m/05s38	Peppermint
/m/016nb4	Pepsin
/m/02x3hw	Pethidine
/m/01nxyd	Petroleum jelly
/m/05dr2v	Phenobarbital
/m/0602p	Phenol
/m/01km9r	Phenolphthalein
/m/0cyvn	Phenytoin

/m/019s9m	Phosphoric acid
/m/025s7x6	Phosphorus
/m/0fp6w	Pineapple
/m/0b3lj	Pistachio
/m/025s7y2	Platinum
/g/120p4gwp	Plum
/m/01b90_	Polio vaccine
/m/071vkz	Polylactic acid
/m/0jwn_	Pomegranate
/m/0frq6	Pork
/m/025s7j4	Potassium
/m/01j604	Potassium hydroxide
/m/03z9vp	Potassium iodide
/m/0h5p_	Potassium nitrate
/m/02fwyz	Potassium permanganate
/g/120j31xn	Potato
/m/02yzx9	Prednisolone
/m/06yvct	Pregabalin
/g/1215jb2v	Progesterone
/m/09pbb	Proline
/m/03xm_b	Promethazine
/m/019dfq	Propranolol
/m/0mzgk	Prostaglandin
/m/0hqw_	Pseudoephedrine
/m/05zsy	Pumpkin
/m/04h5y5	Pyridoxine
/m/09dz_d	Pyrimethamine
/m/0l04c	Pyruvic acid
/m/06b6w	Quinine
/m/06mf6	Rabbit
/g/120jqr76	Radish
/m/04nl3c	Ranitidine
/m/01pd2g	Raspberry
/g/11csqg56b7	Remdesivir
/m/06jry	Riboflavin
/m/09759	Rice
/m/03qsl9	Rifampicin
/m/03l19k	Ritonavir
/m/09g6_4	Rivaroxaban
/m/06cd7	Rosemary
/m/02z705	Rosuvastatin
/m/02ck8_	Salbutamol
/g/120_g7k9	Salicylic acid
/m/075pc	Scopolamine
/m/01hm70	Sea salt
/m/073z8	Serotonin
/m/0136_g	Sertraline
/m/04vp3y	Shea butter
/m/0ll1f78	Shrimp
/m/07_71	Sildenafil
/m/0bylh	Silicon dioxide

/m/025sf8x	Silver
/m/01h2wm	Silver nitrate
/m/014dn0	Sodium bicarbonate
/m/014dnj	Sodium carbonate
/m/0ksgj	Sodium chloride
/m/0fw77	Sodium hydroxide
/m/01cfyd	Sodium nitrate
/m/03clt1	Sodium sulfate
/m/0gyv9	Soybean
/m/016rh7	Spinach
/m/036mpc	Spironolactone
/m/0dv6r	Squash
/m/06x3w	Starch
/m/07fbm7	Strawberry
/m/0fkjv	Sugarcane
/m/0lk81	Sulfate
/m/0dfwj	Sulfur dioxide
/m/077lr	Sulfuric acid
/m/08ct4m	Sunflower oil
/m/0g0y_	Sunflower seed
/m/0dm32	Sweet potato
/m/0dcfv	Table sugar
/m/021rl7	Tadalafil
/m/07qps	Talc
/m/04nv0k	Tamoxifen
/m/08f4cp	Tamsulosin
/m/0gs2h	Tannin
/m/0gll6	Taurine
/m/02_863	Tea tree oil
/m/048xtq	Terbinafine
/m/07m9q	Testosterone
/m/07jvd	Tetracycline
/m/0gjwh	Tetrahydrocannabinol
/m/0gzk2	Theophylline
/m/07hnp	Thiamine
/m/025sk56	Titanium
/m/027yz2q	Tocilizumab
/m/07k6_	Toluene
/m/07j87	Tomato
/m/01l6z8	Tramadol
/m/069sqv	Tranexamic acid
/m/02zcy0	Tretinoin
/m/07jw9	Trypsin
/g/1214fwq8	Turmeric
/m/0cl46	Turnip
/m/07sy1	Urea
/m/07syk	Uric acid
/m/09g33	Valerian
/m/079bqt	Valsartan
/m/07_rn	Vanilla
/m/01gfs2	Vasopressin

/m/0f4l5	Vitamin A
/m/07zqy	Vitamin C
/m/0f4jp	Vitamin D
/m/03bwyyc	Vitamin E
/m/07z_g	Vitamin K
/m/01jjmt	Warfarin
/m/0838f	Water
/m/02d9p2	Watercress
/m/0kpqd	Watermelon
/m/09696	Wheat
/m/025sqqf	Xenon
/m/088lm	Yeast
/m/02dmz4	Yolk
/m/01bqth	Zidovudine
/m/025sqz8	Zinc
/m/02kgj6	Zinc oxide
/m/02hsj4	Zinc sulfate
/m/01_2s3	Zolpidem
/m/01bqth	Zidovudine
/m/025sqz8	Zinc
/m/02kgj6	Zinc oxide

Supplementary Email S1: Email received from Google regarding changes to the Google Health Trends API

Update regarding Google Trends improved data collection

trends-api-support@google.com
To Myburgh, Pieter

Reply | Reply All | Forward

Fri 2022/07/15 23:58

We removed extra line breaks from this message.

CAUTION: This email originated from outside of the University of Johannesburg. DO NOT open any content (links and attachments) if the sender is unknown.

Hi,
you are receiving this email as you were previously granted access to the Google Trends API.

On July 18th, Google Trends will introduce an improvement to its data collection process. With this improvement, Google Trends will be providing higher accuracy responses by using a more comprehensive sample of search requests.

The improvement will apply to data dating back to January 1st, 2022.
Given this, we would kindly ask you to take notice of the following:
- Any sharp increase or decrease in search interest over time around this specific date (Jan 1st 2022) is most likely explained by the said change.
- Month-over-month or year-over-year calculations comparing periods of time before and after Jan 1st 2022 should be reviewed in light of this change.

We understand that this change may be disruptive to your ongoing work with the Trends API. We remain fully committed to providing Trends users with a high accuracy view of what the world is searching for, and this update is an important step in fulfilling this commitment.

Please reach out to trends-api-support@google.com if you have specific questions regarding this change.

Thank you,
The Google Trends Team
